# Neuromodulatory Control and Language Recovery in Bilingual Aphasia: An Active Inference Approach

**DOI:** 10.3390/bs10100161

**Published:** 2020-10-21

**Authors:** Noor Sajid, Karl J. Friston, Justyna O. Ekert, Cathy J. Price, David W. Green

**Affiliations:** 1 Wellcome Centre for Human Neuroimaging, University College London, 12 Queen Square, London WC1N 3AR, UK; k.friston@ucl.ac.uk (K.J.F.); justyna.ekert.14@ucl.ac.uk (J.O.E.); c.j.price@ucl.ac.uk (C.J.P.); 2 Experimental Psychology, University College London, Gower Street, London WC1E 6BT, UK; d.w.green@ucl.ac.uk

**Keywords:** bilingual aphasia, active inference, generative models, simulation, in-silico lesions, neuromodulatory control, language recovery patterns, alternate antagonism, paradoxical translation

## Abstract

Understanding the aetiology of the diverse recovery patterns in bilingual aphasia is a theoretical challenge with implications for treatment. Loss of control over intact language networks provides a parsimonious starting point that can be tested using in-silico lesions. We simulated a complex recovery pattern (alternate antagonism and paradoxical translation) to test the hypothesis—from an established hierarchical control model—that loss of control was mediated by constraints on neuromodulatory resources. We used active (Bayesian) inference to simulate a selective loss of sensory precision; i.e., confidence in the causes of sensations. This in-silico lesion altered the precision of beliefs about task relevant states, including appropriate actions, and reproduced exactly the recovery pattern of interest. As sensory precision has been linked to acetylcholine release, these simulations endorse the conjecture that loss of neuromodulatory control can explain this atypical recovery pattern. We discuss the relevance of this finding for other recovery patterns.

## 1. Introduction

Lesions induced by stroke or by head injury in speakers of more than one language elicit a variety of recovery patterns, e.g., [1,2]. We lack effective accounts of these patterns, yet an understanding of their mechanistic basis is theoretically and practically important. Theoretically, recovery patterns require an understanding of their neurocomputational bases in order to explain the variety. Practically, such approaches may inform and enhance personalised treatments [3]. In our view, fundamental to reaching an adequate account is recognising that brain-damaged individuals are agents who are trying to make sense of their world and act as effectively as they can towards that goal. We first describe some key patterns of recovery. We then provide a non-technical description of our approach to understanding these patterns, based upon computational neuropsychology and active inference [4].

### 1.1. Recovery Patterns and Control

Neuropsychological case reports of language recovery in bilingual speakers document instances best characterised as indicative of intact language networks with impaired control. For instance, S.J., a Friulian-Italian speaker, had intact clausal processing in both languages but in conversation was unable to avoid switching inappropriately into Friulian, for example, when speaking to an Italian-only speaker [5]. Problems controlling relatively intact networks also offers a parsimonious way to account for selective recovery in which one language, but not the other, is recovered—and for instances where one language recovers but the other becomes progressively impaired (for review, [6]). Indeed, effective connectivity studies corroborate such a claim [7]. Language control regions—see [8] for a review—engaged during picture naming revealed increased effective connectivity with the language network post-treatment for the treated language and decreased connectivity for the untreated language. Such alterations in connectivity are thought to mediate shifts in performance. Problems of language control are also relevant to understanding parallel language recovery. In parallel recovery, post-stroke performance is in line with pre-morbid self-ratings of proficiency in each language. With this pattern of recovery, patients have difficulty in controlling verbal interference (e.g., as indexed by conflict in a verbal Stroop task) especially in their non-native language [9].

Consistent with this separation of language networks and their control—but particularly challenging from an explanatory point of view—are case reports of individuals with a pattern of alternate antagonism and paradoxical translation. Paradis et al. [10] reported two such cases. We briefly describe one here. A.D., a 48-year-old nun fluent in French and Arabic, suffered a left temporoparietal contusion after a moped accident in Morocco. After an initial period of complete aphasia, followed by a period of speaking a few words of Arabic, she was flown to a hospital in France and evinced the recovery patterns at issue. Alternate antagonism refers to the fact that on one day A.D. used first language (L1) spontaneously (and clinically named pictures in that language) but was unable to use the other language (L2). On the following day, the reverse pattern obtained with use of L2 but not of L1. This pattern of alternate antagonism was accompanied by good comprehension in both languages and the ability to repeat words normally in L1 and in L2. In addition, her recovery showed paradoxical translation, as she could not translate into the language used for naming but could translate into the language that she could not use. Evidently such a pattern of recovery precludes destruction or isolation of language networks. In terms of classical neuropsychology [11], A.D. exhibits a “double-dissociation” between naming and translating.

Green [12] offered a narrative account of her itinerant pattern of recovery. A hierarchical control structure (elaborated subsequently in [13]) specified how particular tasks such as naming or translating were performed and the resources (putatively, neuromodulators) required to exercise such control. Naming a picture in one language entails suppressing a response from the other language network, i.e., external suppression of the non-target network. By contrast, translating into a language entails suppressing the repetition of the word to be translated, i.e., internal suppression of that network. Constraints on the resources recruited by a particular task lead to a loss of control: by way of illustration, insufficient resources for external suppression of an L2 network would block naming of a picture in L1. However, with sufficient resources to suppress repetition of an L2 word to be translated (internal suppression of the L2 network) translation into L1 is possible. In this formulation, the use of a resource for a particular task (e.g., the external suppression of network outputs; or the internal suppression of repetition within a network) depletes the resource for that task. The trajectory of recovery then reflects the task-specific depletion and regeneration of resources that suppress or select networks in a given task context (i.e., maintain task set and appropriate language processing). We pick up this central notion when simulating naming and translation in silico.

To simulate the recovery patterns of alternate antagonism and paradoxical translation, we introduce in-silico lesions to a synthetic subject engaged in active inference, described in detail in Section 2. We use three tasks that evince the recovery patterns above: picture naming (i.e., seeing a picture and naming it), word repetition (i.e., hearing a word and repeating it back in the same language) and translating a heard word (i.e., repeating it back in the other language). In this setting, the in-silico lesions disrupt the ability to select appropriate actions to complete each task (e.g., to name a picture in L1).

### 1.2. Active Inference, Generative Models and Bayes-Optimality

Active inference provides a first principles, Bayesian, account of how agents actively engage with a given environment [14,15,16]. Central to this approach is the notion of a generative model. Such a model is an abstraction and embodies an agent’s hypotheses about the state of the sensed world. The objective of active inference is to calibrate the generative model so that it best explains the agent’s sensory observations, thereby minimising surprise (as scored with variational free energy) and reducing their uncertainty about the environment (as scored by expected surprise or free energy in the future).

This Bayesian approach is formally distinct from previous computational approaches to aphasia [3,17,18,19,20]. For example, in [3]—in an extension of the DISLEX model [21]—self-organising maps were used to account for treatment effects. This kind of modelling usually requires a training phase to make appropriate predictions. In contrast, our approach provides a complementary perspective. No ‘training’ procedure is necessary; instead, outcomes are simulated based on learnt, or pre-specified, prior beliefs based on the generative model architecture. In other words, we focus on language processing as a form of inference, as opposed to learning. This can be very useful for understanding behavioural deficits arising from neurological damage [4,22,23].

Under active inference, we are concerned with what agents must believe to render their behaviour optimal, instead of why it appears pathological. This allows us to characterise patients with brain damage as operating under ideal Bayesian assumptions but with a poor (i.e., lesioned) generative model [4,23]. In other words, one can characterise abnormal behaviour as (Bayes) optimal inference under abnormal prior beliefs inherent in a patient’s generative model. Technically, we simulate language processing as Bayesian belief updating using (simulated) neuronal dynamics that perform a gradient descent to minimise variational free energy, which implicitly maximises model evidence, i.e., the evidence for the patient’s generative model of the world. This is sometimes referred to as self-evidencing [24]. We reserve description of technical details for Section 2 (Methods).

The in-silico lesions used in this work are motivated by the neuromodulatory control account described above. These lesions alter the confidence (or precision) in prior beliefs about the causes of sensory observations. These changes in precision can be linked to (a loss of) neuromodulatory control, and to acetylcholine in particular [25,26,27]. In the next Section 2 (Methods), we provide a detailed introduction to active inference, our generative model and the simulation procedure involved. In the section that follows, Section 3 (Results), we present these simulations of alternate antagonism with paradoxical translation. The simulations provide proof of principle that aberrant precision control (i.e., neuromodulation) can explain this recovery pattern and, crucially, emerge naturally from a Bayes-optimal response to brain damage. These simulations can therefore be read as a proof of principle that loss of neuromodulatory control can underwrite observed patterns of recovery. Section 4 (Discussion) discusses the findings, the neuromodulatory interpretation, and the wider implications of the active inference approach.

## 2. Methods

Our simulations were carried out using an active (Bayesian) inference model of three tasks: picture naming, word repetition and translation. In this section, we first provide a concise overview of active inference and then present the generative model used for the simulations. Please see Table 1 for a glossary of the terms used.

### 2.1. Active Inference

Active inference brings together perception and action under a formal, first principles, Bayesian framework [14,15,16]. It postulates that all sentient creatures minimise free energy (F) [28] or maximise model evidence [29,30]. Here, free energy is simply the complexity cost incurred in forming accurate posterior beliefs about causes of sensation:

(1) $$F=\underset{\mathrm{complexity}}{\underset{︸}{D_{KL}[Q(s)||P(s)]}}-\underset{\mathrm{accuracy}}{\underset{︸}{E_{Q(s)}[\log P(o|s)}}]$$

Under active inference, sentient creatures not only have capacity to infer the state of the world but can also influence the future (i.e., make the environment less surprising) by taking actions. These actions are selected from trajectories of all plausible actions (i.e., policies, π), by minimising their expected free energy (G) [31,32] in accordance with the principle of least action.

The expected free energy can be derived from the free energy by taking an expectation under predicted outcomes in the future *P*(*o_τ_*|*s_τ_*):

(2) $$G(\pi )=\underset{\mathrm{Risk}}{\underset{︸}{E_{\tilde{Q}}[\log(Q(s_{\tau }|\pi ))-\log(P(s_{\tau })]}}\underset{\mathrm{Ambiguity}}{\underset{︸}{-E_{\tilde{Q}}[\log(P(o_{\tau }|s_{\tau }))]}}$$

where $\tilde{Q}=P(o_{\tau }|s_{\tau })Q(s_{\tau }|\pi )$ and $Q(o_{\tau }|s_{\tau },\pi )=P(o_{\tau }|s_{\tau })$ From Equations (1) and (2), it can be seen that expected complexity corresponds to risk (the difference between predicted and preferred futures), while ambiguity is the expected inaccuracy.

From this free energy formulation, we can optimise expectations about hidden states, policies and precision through inference and optimise the model parameters through learning. This involves the (variational) message passing of sufficient statistics of posterior beliefs among neuronal populations. Here, the sufficient statistics are just the expected probability of being in a particular state or pursuing a particular policy (i.e., sequence of actions). Variational message passing can be formulated as a gradient descent on variational free energy, using a mean-field approximation [33,34]. This has been shown to provide a plausible account of neuronal dynamics [14,35,36].

The process theory underwriting active inference is based on a partially observable Markov decision process (POMDP). This can be defined as a generative model which, in its simplest form, has discrete outcomes that are caused by discrete hidden states, and is described extensively in previous work [14,37]. A generative model can be decomposed as:

(3) $$P(o,s,\pi ,\eta )=P(\pi |\eta )P(\eta )\prod_{t=1}^{T}P(o_{\tau }|s_{\tau },\eta )P(s_{\tau }|s_{\tau -1},\pi ,\eta )$$

where η are model parameters, over outcomes, states and policies.

In this generative model, outcomes (i.e., sensory observations) depend upon hidden states and hidden states depend upon policies. Outcomes and hidden states are generated by two sets of categorical probability distributions parametrised by A and $B_{\pi }^{}$. The first distribution is the likelihood, A, which maps hidden states to outcomes. The second is associated with the probabilistic transitions, $B_{\pi }^{}$, among the hidden states, under policy *π*.

Outcomes are generated by selecting appropriate policies using a softmax function of their expected free energies. Therefore, policies are more probable, a priori, if they minimise the expected free energy, which depends upon prior preferences about outcomes. From this, sequences of hidden states are generated using state transitions specified by the selected policy. These hidden states generate outcomes. The hidden states can also influence the expected free energy. Here, model parameters are equipped with a prior (categorical) distribution.

This model specification may sound rather technical and complicated; however, it is the simplest and most generic kind of generative model for deeply structured state transitions of the sort required to generate behaviours such as speech. The form of this generative model can be considered the ‘structure’ in structure–function relationships. This structure is thought to underwrite functional brain architectures that realise active inference; e.g., [38]. This perspective allows us to associate in-silico lesions with anatomical lesions. When formulating model inversion in terms of neuronal message passing, the likelihood A mapping takes the form of extrinsic connections (i.e., between different neuronal populations encoding posterior expectations) while the transition B mapping takes the form of intrinsic connections (i.e., connectivity within neuronal populations encoding posterior expectations over time) [35].

### 2.2. Generative Model of Picture Naming, Word Repetition and Translation

Our aim was to illustrate how alternate antagonism with paradoxical translation could be mediated by a loss of sensory precision, and an implicit loss of neuromodulatory control. For this purpose, we modelled three language tasks using a minimal generative model: (1) picture naming, where the subject is presented with a visual stimulus and must verbally identify it [39], (2) word repetition, where the subject repeats a heard word in the same language [20,40,41,41,42] and (3) word translation, where the subject repeats a heard word in another language [43]. These tasks are apt, since they allow us to simulate behaviours open to bilingual speakers: single language use (picture naming and word repetition) and use of both languages (word translation) behaviour and more specifically, target the type of instrument used in clinical assessments. Here, the model probability distributions that underwrite Bayesian belief updating are based on an empirical understanding of how subjects respond during these three different language tasks. In other words, we assume that real subjects adopt formally similar generative models.

**Figure 1:**
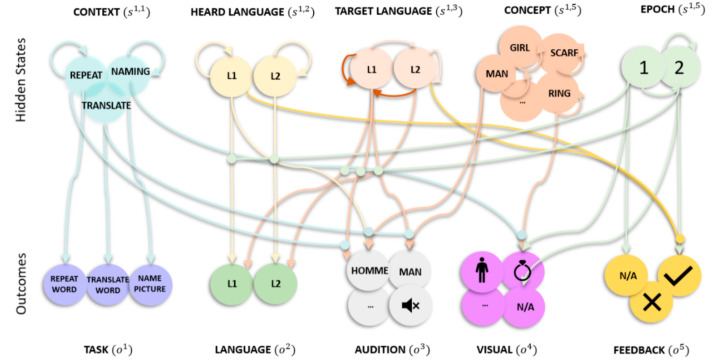
(generative model) Graphical representation of the picture naming, word repetition and translation tasks. There are five outcome modalities: task, language, audition, visual and feedback, and five (hidden) state factors with the following levels (i.e., possible alternative states). Context (3 levels) indexes the current task (naming, repetition, translation). The heard language factor (2 levels) lists the languages the experimenter can use to test the participant (L1 or L2). The target language factor (2 levels) lists the languages the participant can respond in (L1 or L2). The concept factor (12 levels) lists the concepts that the experimenter can ask the participant to name, repeat or translate. The epoch (2 levels) indexes the phase of the trial (stimulus presentation, response and feedback). The lines from states to outcomes represent the likelihood mapping and the lines mapping states within a factor represent allowable state transitions. To avoid visual clutter, we have highlighted likelihoods and transition probabilities that are conserved over state factors and outcome modalities. For example, in the audition likelihood mapping for translation, the target language and concept (man) to audition (homme) is shown for epoch 1, but similar mappings apply when mapping between girl and fille, etc. One (out of two) example transition probability is highlighted (in darker brown shade) for the target language, i.e., the transition is always to L1, regardless of previous target language. This transition represents the choice to speak in L1. Similar mappings are applied when choosing to speak in L2, regardless of the previous state.

The generative model has one level with five (latent or hidden) state factors: context, target language, heard language, concept and epoch, along with five outcome modalities: task, language, audition, visual and feedback (Figure 1). The context factor has states that correspond to the three different tasks: naming pictures, repeating and translating words. The heard language has states corresponding to the heard language: L1 and L2. The target language factor’s states covered what language the reply should be in: L1 and L2. The concept factor has 12 states, corresponding to the concept in play: man, girl, baby, ring, scarf, hat, cat, dog, parrot, leaflet, book, and newspaper—regardless of language. The epoch factor covers stages of the trial: listening to or seeing the stimulus (i.e., epoch 1), responding to the stimulus (i.e., epoch 2) and receiving performance evaluation (i.e., epoch 2). In terms of outcome modalities, the task outcome reports what the current task is: picture naming, word translation or repetition. The language outcome reports the current (spoken or heard) language: L1 or L2. The audition outcome reports the current (spoken or heard) word. We exemplified with English and French words (but words of other languages could have been used with no implications for the simulation): man, homme, girl, fille, baby, l’enfant, ring, bague, scarf, écharpe, hat, chapeau, cat, chatte, dog, chienne, parrot, perroquet, leaflet, brochure, book, livre, newspaper, la feuille or N/A. The visual outcome reports the picture shown: man, girl, baby, ring, scarf, hat, cat, dog, parrot, leaflet, book, newspaper or N/A. The evaluation outcome represents the positive or negative response received (only provided at the second epoch). In Figure 1, the lines represent plausible connections (and their absence reflects implausible connections), with the arrow denoting direction. For example, the line mapping hidden state epoch ‘1’ to outcome modality feedback ‘N/A’ suggests that ‘N/A’ is only plausible at epoch ‘1’, but not ‘2’. Similarly, the line for hidden state concept girl to itself reflects that level girl can only transition to itself and no other concept, throughout the trial.

The likelihood, A, is represented by the lines connecting states to outcomes in Figure 1 and each outcome modality is associated with its own likelihood. The task likelihood depends on the context factor; if I believe that I need to name a picture then the task is picture naming. Likewise, if I believe that I need to repeat (translate) words, then the task is word repetition (translation). The language likelihood depends on the epoch, target and heard language factor: if I believe it is epoch 1 and I am listening to the task instructions, and the target language is L1 (L2), then I can hear L1 (L2)—irrespective of what the target language is. Conversely, if I believe it is epoch 2 and the heard language is L1 (L2), then I am speaking L1 (L2), irrespective of what the heard language is. The audition likelihood depends on either the heard language and concept (epoch 1) or the input target language and concept (epoch 2) factors. That is, the generated audition outcomes are determined by the current epoch, e.g., during epoch 1, the audition likelihood maps the heard language (L1) and concept (man) to auditory input (homme). The visual likelihood is defined as a one-to-one mapping between the concept and visual input if I believe I am at epoch 1 of the trial and mapped to N/A for epoch 2. The feedback likelihood depends on all the hidden states. Positive evaluation is given at epoch 2: if I am naming, repeating or translating the previously presented (auditory or visual) stimuli correctly. For example, if I am repeating homme, after hearing homme during a word repetition task, I will get positive feedback. The likelihood is defined as mapping to (i) neutral feedback—regardless of target and heard language—if the epoch is 1, (ii) positive feedback, if the heard and target language match at epoch 2 for a picture naming or word repetition task, (iii) positive feedback, if the heard and target language do not match at epoch 2 for a word translation task, and (iv) negative otherwise.

The transition matrices, B, are represented by lines modelling transitions among states within each factor in Figure 1. The transition matrix for context, heard language and concept factor is an identity matrix. This means they stay the same across all epochs. For the target language factor, there are two possible transitions. These involve transitions to a specific language where the language depends upon which action is selected. An example transition would be that when I choose to speak in L1, regardless of previous language (L1 or L2), I transition to L1 (highlighted in Figure 1 with brown transition lines). Accordingly, target language is the control state that allows the model to take actions that impact the environment. For the epoch factor, the transitions are from 1 to 2, with 2 being an absorbing state (i.e., the final epoch is of the second epoch type).

The model was allowed to choose from a set of 2 different one-step policies (sequences of actions); both are a different permutation of how (controlled) state transitions might play out. The prior beliefs about the initial states were initialised to 1 for all repeated and target word levels, epoch 1 for the epoch factor and zero otherwise. The model had no capacity to learn between each simulation. Additionally, certain aspects of this generative model and the optimisation process (i.e., variational message passing scheme) can be mapped onto the functional anatomy in the brain, e.g., states (circles in Figure 1) can be associated with neuronal populations and functions (lines in Figure 1) can be associated with neuronal connections along which messages (e.g., action potentials) are passed [14]. Consequently, we can formulate hypothesis-driven assignment of states and outcomes to particular neuronal populations in particular cortical and subcortical structures or, indeed, within the cortical lamina of canonical microcircuits. Please see Friston et al. [14] for further details and references.

### 2.3. In-Silico Lesions

To simulate alternate antagonism and paradoxical translation, we introduced in-silico lesions to the model of picture naming, word repetition and translation described above (Figure 1). This involved perturbing precision over the generative model parameter A—resulting in structural (i.e., synaptic) changes that affect the optimisation procedure (i.e., belief updating). Here, the parameter A maps outcomes given their causes, and in doing so couples adjacent (cortical) levels of the generative model. These structural assumptions mean that A can be associated with extrinsic (between region) connectivity and lesions to these connections reproduce disconnections similar to those seen by destruction of white matter tracts and/or any pathology associated with projection neurons, including axonal (white matter) lesions.

**Figure 2:**
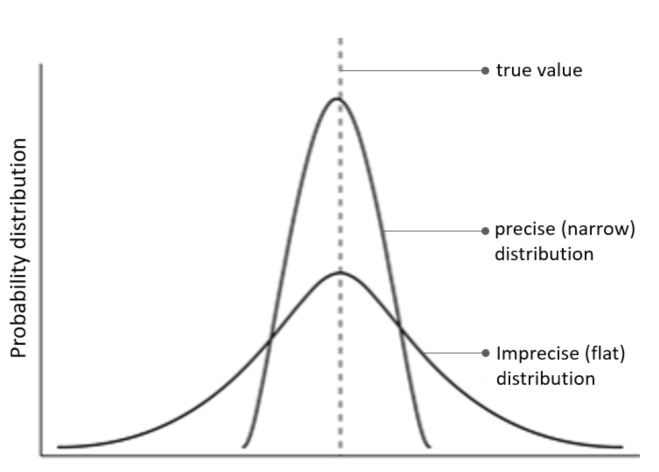
(precision) The figure plots two hypothetical probability distributions—one precise (narrow) and the other imprecise (flat).

The in-silico lesions are introduced by shifting the precision, ω, control over A. Precision—a hyperparameter of the model—is the inverse uncertainty over parameter A and influences the confidence in beliefs. For intuition, we plot two (hypothetical) probability distributions: a precise distribution which has confident beliefs centred around the true value, and an imprecise distribution which implies flatter beliefs about the true value (Figure 2). In terms of A, precise beliefs entail the model being confident that a particular stimulus (outcome) was generated by a particular language (cause). Conversely, imprecise distributions imply ambiguous association between causes and outcomes, and observations do little to resolve this uncertainty (i.e., increased prediction errors). Thus, A precision corresponds to the confidence with which causes can be inferred from observations.

For our simulations, perturbing precision control was implemented using discrete updates to the precision hyperparameter, ω, over A until saturation. This mimics precision control—regulated by higher levels of the model hierarchy—where belief updates influence parameter uncertainty. Neurobiologically, changes in the precision of beliefs about outcomes given states of the world (A) is altered by acetylcholine [25,26,27]. As a neurotransmitter, acetylcholine controls sensory activities that depend on selective attention [44,45], and arguably enable bilingual speakers to select the appropriate language (see Section 4 Discussion).

### 2.4. Paradigm Procedure

**Figure 3:**
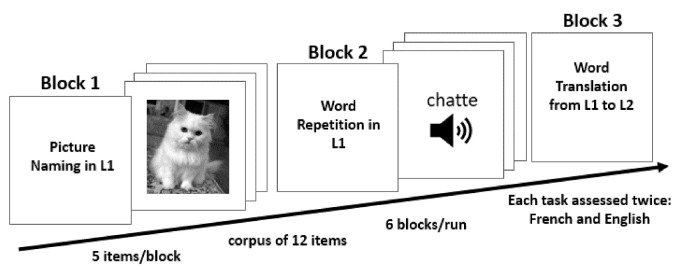
(paradigm set-up) Schematic illustration of the task sequence the model was exposed to during each simulated day.

Both our model simulations—lesioned and control—were exposed to the same paradigm procedure each day (Figure 3). This included six consecutive experimental blocks in the following order: picture naming in L1, word repetition in L1, word translation from L1 to L2, picture naming in L2, word repetition in L2 and word translation from L2 to L1. Each block consisted of 5 items chosen from the corpus of 12 concepts: man, girl, baby, ring, scarf, hat, cat, dog, parrot, leaflet, book, and newspaper.

## 3. Results

In this section, we describe the simulation of alternate antagonism and paradoxical translation recovery patterns in bilingual aphasia based on the patient cases reported in [10] and summarised in Table 2. Word repetition was intact throughout and is not detailed in Table 2.

We simulated these recovery patterns using an active inference under a generative model of the three tasks: picture naming, word repetition and translating a heard word (Section 2). We distinguish between the heard language, target language, context (i.e., the task) and the stimulus (i.e., word or picture) and introduce in-silico lesions by altering the precision of the mapping to sensory outcomes from these latent causes that the agent has to infer. This is also known as sensory precision; namely, the precision of the likelihood mapping between (latent) causes and (sensory) consequences. Perturbing the precision of this mapping (parameter A in the model) led to a quantitative disconnection between the words heard by the synthetic subject and posterior beliefs about their possible causes. This partial disconnection decreased the posterior confidence over the causes of what the subject heard and consequently rendered belief updating less precise. The locus of this in-silico lesion was motivated by the fact that bilinguals must be able to select the target language and adjust their own speech with respect to it. We comment further on this point in the Discussion (Section 4).

**Figure 4:**
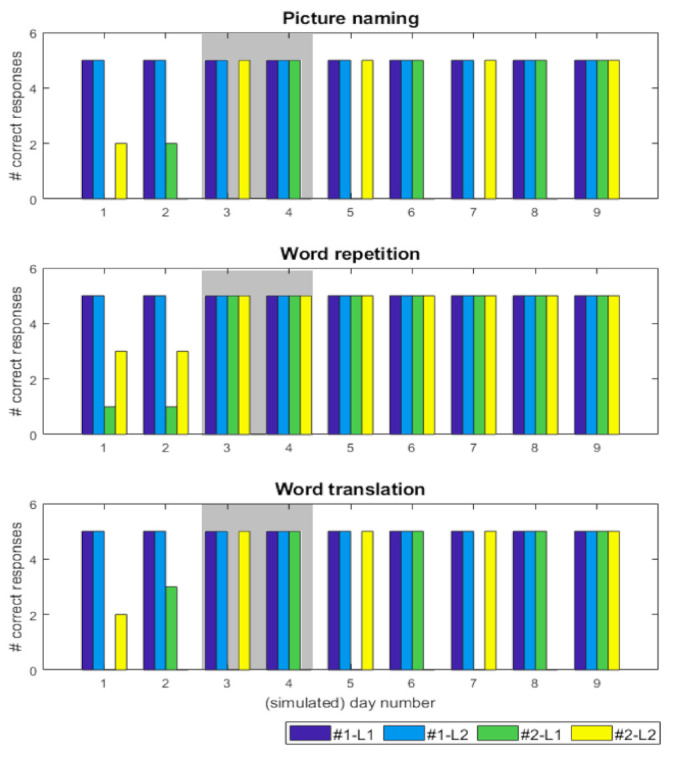
(simulated behavioural performance) The bar charts report the number of correct responses, for each task, across the 9 simulated days for two simulated subjects. Here, #1 is a control subject (dark and light blue bars) and #2 is a lesioned subject (green and yellow bars). The language (L1/L2) in the legend denotes the speaker language e.g., for #1-L1 the subject was asked to repeat the word in L1 but translate the word from L1 to L2. Similarly, for #2-L2 the object had to be named in L2 but translated from L2 to L1 during the translation task. The x-axis is the simulated day number and the y-axis denotes the number of correct responses. The maximum number of correct responses, for each task per day, is 5. Day 3 and 4, in the grey box, is highlighted, the pattern of alternate antagonism and paradoxical translation in subject #2, e.g., when L2 is accessible (yellow bars; day 3), the subject is unable to name pictures and translate from L1 but can repeat in both L2 and L1. However, day 4 shows an alternate recovery profile: L1 is accessible (green bars), the subject is unable to name pictures and translate from L2 but can repeat in both L2 and L1.

Simulation of the recovery pattern was accomplished with discrete changes in sensory precision ω; i.e., 0.1, 0.25, 0.5, 1, that alternated between the heard language affected. These changes limit the subject’s ability to select the appropriate language. Over time, these failures of inference resolve—as precision increased—to rebalance control over processing in both languages. Figure 4 shows the behavioural characterisation of the simulation of the recovery pattern of the first patient (A.D.) [10]. Similar precision updates, at a slower timescale, would reproduce the recovery pattern for the second patient (Table 2).

The responses of a lesioned subject [46]—and a control (no lesion) subject (#1)—were simulated for 9 days with 30 trials each day (based on random initialisation seeds). See Section 2.4 for exact details of the simulation set-up. By using the same initialisation seeds we test for specific counterfactuals, i.e., had it not been for the lesion, the control and this subject would have performed in exactly the same way.

As shown in Figure 4, the control subject had 100% correct responses for each task, for both languages. Conversely, the lesioned subject demonstrates a fluctuating recovery pattern after the in-silico lesion, with the available language for naming alternating for consecutive periods, until it stabilises. In short, the performance differences, over the 9 simulated days, are caused by the fluctuating changes in (sensory) precision during the belief updating process. Specifically, when precision is perturbed, it compromises the ability to infer the precise cause of observed outcomes and appropriately propagate this information to the future in order to plan a response. This is because at each trial, the subject has beliefs about both the heard language and the target language. However, when the precision is reduced, this introduces uncertainty about plausible causes of outcomes and accordingly affects the agent’s ability to update beliefs and plan her actions appropriately.

**Figure 5:**
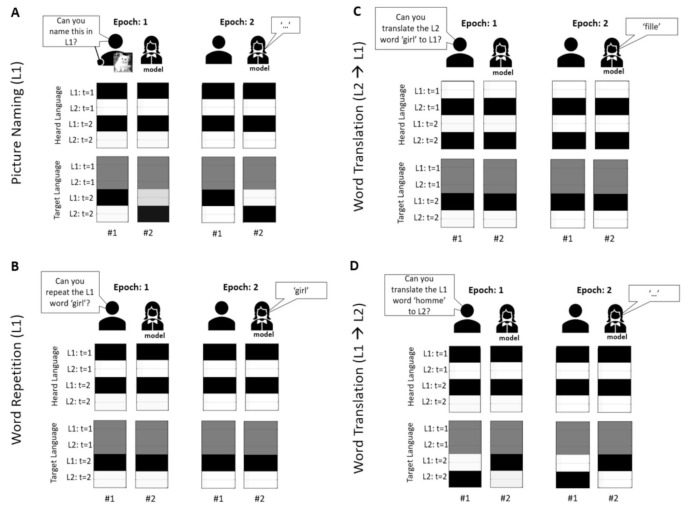
(belief updates) Each figure (**A**–**D**) reports belief updating, over two epochs of a single trial, for the two subjects: #1-control and #2-lesioned. These are shown for the *target language* and *heard language* factors when completing different tasks during the simulated day 3, when the model had access to L2. These tasks are picture naming in L1 (**A**), word repetition in L1 (**B**), word translation from L2 to L1 (**C**) and word translation from L1 to L2 (**D**). For each figure, the top row comprises an image showing the stage of the trial and the bottom two rows display the belief updates for heard and target language. The columns represent the two epochs for the two models. The first column represents the posterior expectations about each of the associated states at different epochs (current, future) at epoch 1, for subjects #1 and #2. Similarly, the second column represents the posterior expectations about each of the states at different epochs at epoch 2. For example, for the target language factor, in both panels there are 2 states, and a total of 2 × 2 (states times epochs) posterior expectations. Similarly, for the heard language factor there are two levels, and total of 2 × 2 expectations. Note that white represents an expected probability of zero, black of one, and grey indicates gradations between these extremes. For example, the first column, top row in A corresponds to expectations about the *heard language* in terms of two alternatives for the first epoch. The second column, top row reports the equivalent expectations for the second epoch. This means that at the beginning of the trial the second column reports beliefs about the future; namely, the next epoch. However, later in time, these beliefs refer to the past, i.e., beliefs currently held about the first epoch as seen in the second column. This aspect of (deep temporal) inference is effectively an implementation of working memory that enables our model to remember what it has heard—and accumulate evidence for the *target language* that is subsequently articulated; i.e., mediating a working memory for planning short-term responses. Note that most beliefs persist through time. For example, the *heard language* reveals itself almost immediately and this prospective belief is propagated into the future.

To make this concrete, the impaired belief updating is shown in Figure 5. Here, when the lesioned subject [46] has impaired access to L1 during a picture naming task—due to a decline in precision—she is unable to correctly infer the target language at epoch 1 and ends up replying incorrectly (in L2) (Figure 5A). Similarly, during a translation task from L1 to L2 (Figure 5D), the subject is unable to infer that L2 is the target language and also replies incorrectly at epoch 2 in L1. In contrast, the target language, during word repetition in L1 (Figure 5B) and word translation from L2 to L2 (Figure 5C), reveals itself almost immediately and these prospective beliefs are propagated into the future, with correct responses at epoch 2.

The data presented above were simulated using a generic optimisation scheme implemented using standard functions. These are available under the SPM academic software: www.fil.ion.ucl.ac.uk/spm/. Specifically, the code (and simulated data) necessary to reproduce the simulations and figures can be found here: www.github.com/ucbtns/aapt.

## 4. Discussion

Bilingual patients reveal a variety of patterns of language recovery. Our goal was to explore the putative neurocomputational basis of one complex pattern (alternate antagonism with paradoxical translation) with the hope of identifying the factors that might elicit other patterns of recovery. We build on a symbolic hierarchical control model [12,13] that distinguishes processing in language networks from the control and selection of these networks. Experimental research suggests that complexity of the control process is predictive of behavioural and ERP data (e.g., [47]) and that complexity can inform treatment [48]. Loss of control, specifically, constraints on neuromodulatory resources, is proposed to mediate recovery patterns. The simulations of belief updating reported in this paper provide formal support for this conjecture—because we were able to reproduce the behavioural phenomenology with a single manipulation; namely, the precision of the mapping between (unobserved) states of the world and (observed) sensory input. In the active inference literature, this precision is thought to depend on the neuromodulator acetylcholine.

The tenet of our approach is that behaviour has to be understood from the perspective of agents acting in their worlds. This tenet is embodied in the active inference (Bayesian) approach to understanding behavioural deficits following on neurological damage. Central to the approach is the notion that action in the world is underwritten by the agent’s model of how the world generates her sensations. The objective—in active inference—is to adjust the generative model, so it provides the best explanation of sensory observations, thereby minimising surprise and reducing uncertainty about the present and future states of the world.

To simulate the recovery pattern we lesioned an in-silico active inference agent and probed her responses to three key tasks: picture naming (i.e., seeing a picture and naming it), word repetition (i.e., hearing a word and repeating it back in the same language) and translating a heard a word (i.e., repeating it back in the other language). Our in-silico lesion reduced the model’s ability to select an appropriate action (e.g., to name a picture in L1). It did so by altering confidence, that is, precision, in prior beliefs about the causes of a sensory observation such as hearing a word in L1. Before this sensory precision stabilised, the synthetic subject exhibited a pattern of alternate antagonism, combined with paradoxical translation with repetition remaining essentially intact. Our simulations accordingly provide a proof in principle that aberrant precision control can explain a complex pattern of recovery seen in neuropsychology. The simulation is agnostic with respect to details of neural implementation. However, in line with [12] that loss of neuromodulatory control may mediate the recovery pattern, changes in precision control have been associated with a specific neuromodulator, acetylcholine, on several lines of evidence [27].

### 4.1. Neuromodulation and Precision

Neuromodulators exert widespread influence on neural networks in the brain. Recent research [49] establishes a direct connection between precision and acetylcholine (ACh) release. ACh enhances sensory precision (e.g., by boosting bottom-up auditory signals) enabling the brain to respond optimally. Enhancing sensory precision is likely to be important in the auditory world of bilingual speakers, as bilinguals must be able to select the relevant target language and adjust their speech accordingly. Research provides evidence of an adaptive response to demands faced by bilingual speakers in their environments. Fundamental pitch (F0) provides a cue for identifying language. This pitch is encoded in the auditory brainstem, whose responses to changes in fundamental pitch are significantly enhanced in bilingual compared to monolingual speakers [50,51]. In the present context, we note that the brain stem is also a source of ACh release (from the nucleus basalis of Meynert). Corroborating its role in attentional control, bilingual speakers show enhanced performance on tests of sustained attention, with performance correlating strongly with brainstem responses in a multi-talker context [50,51]. This coupling of auditory responses and attentional control suggests that use of more than one language modifies top-down influences on sensory processes, perhaps via Hebbian learning [52]. This motivates the pharmacological study of recovery patterns with a view to determining the role of ACh in recovery patterns and precision control, as per [49].

### 4.2. Generalisation and Limitations

The findings we report concern just one pattern of recovery—alternate antagonism and paradoxical translation (Table 2). However, changes in precision may explain other patterns of recovery. Gradual changes in precision may mediate the parallel recovery of both languages where issues of control also arise [9]. Non-parallel recovery patterns (e.g., [53,54]) such as selective recovery and antagonistic recovery may reflect impaired precision control associated with a single language.

Our simulations involved explicit manipulation of precision—mimicking fluctuating precision control—regulated by some higher levels of the model hierarchy. We acknowledge that a more dynamical account is possible in a more expressive generative model. For this, we could go in one of two ways—either include continuous lower level parameters, responsible for speech production including the fundamental pitch (F0), [55] or discrete parameters at the higher level [33] in the model hierarchy. Precision could have also been introduced as a continuous parameter within the current generative model but this would have entailed a slightly more involved belief updating scheme [14]—not part of the (well-established) SPM software.

Changes in precision are sufficient to account for a complex pattern of recovery but our simulation does not establish that they are necessary. Other manipulations within the generative model may be equally successful in explaining similar recovery patterns. For example, one could manipulate priors over target language state transitions. That is, how do an agent’s posterior beliefs about the current target language shape their beliefs about the future. Such manipulations would directly affect language control and might be associated with noradrenaline release, cf. [27].

Regardless of the outcomes of further research, the simulations we report establish the importance of understanding how language networks are controlled in order to understand recovery patterns in bilingual speakers. Furthermore, they may be relevant to language recovery in speakers of a single language, where network control is increasingly acknowledged as crucial for understanding language recovery [56].

## 5. Conclusions

Our active (Bayesian) inference approach offers a different (complementary) perspective to other computational approaches, which is particularly useful for understanding behavioural deficits arising from neurological damage. This approach defines computational pathologies, underwritten by abnormal prior beliefs, that are a Bayes-optimal response to damage. We demonstrated this by lesioning an in-silico active inference generative model and established that variations in confidence in prior beliefs (precision) about the causes of a sensory observations (such as hearing a word) were sufficient to explain the recovery pattern of alternate antagonism and paradoxical translation. Neurobiologically, changes in precision have been linked to the neuromodulator acetylcholine. As such, our data support the notion that constraints on a neuromodulatory resource mediate a loss of language control, whereas stabilising that resource yields normal performance.

## Figures and Tables

**Table 1:** Generic terms used in active inference (definitions).

Term	Description
Probability distribution, P(.)	The probability of a random variable taking a particular value.
Variational distribution, Q(.)	An approximate posterior distribution (i.e., Bayesian belief) over the causes of outcomes, given those outcomes.
Hidden states, s∈S	Latent or hidden states of the world generating outcomes.
Outcomes, o∈O	Outcomes or (sensory) observations.
Action, u∈U	A (control) state that can influence states of the world.
Policy,π∈∏	Sequence of actions.
Generative model, P(o,s)	A joint probability distribution over hidden states and outcomes.
Free energy, F	An information theory measure that bounds the surprise when sampling and outcome, given a generative model.
Complexity, $D_{KL}[Q(s)||P(s)]$	A measure of how much the posterior beliefs have to move away from prior beliefs to provide an accurate account of sensory data.
Accuracy, $E_{Q(s)}[\log P(o|s)]$	The expected log likelihood of the sensory outcomes, given some posterior beliefs about the causes of those data.
Expected free energy, G	Free energy expected under future outcomes—an uncertainty measure, associated with a particular policy.
KL-Divergence, $D_{KL}[.||.]$	A measure of how one probability distribution differs from a second, reference probability distribution.
Temporal horizon, τ∈T	Number of timesteps in a sequence of actions, i.e., policy depth.
Posterior	Beliefs about their causes of outcomes after they are observed. The products of belief updating.
Prior	Beliefs about the causes of outcomes before they are observed. A likelihood and prior beliefs constitute the generative model.
Likelihood, $P(o_{\tau }|s_{\tau },\eta )$	Probabilistic mapping between states and outcomes.
Transitions, $P(s_{t}|s_{t-1},\pi )$	Probabilistic transitions from one state to another over time.
Expectation, E[.]	The average of a random variable.
Precision, ω	Confidence or inverse uncertainty.
Sufficient statistics	Quantities which are sufficient to parameterise a probability distribution.
Gradient Descent	An optimisation scheme used to minimise a particular function by iteratively moving in the direction of steepest descent.
Softmax function, σ	A function that converts a set of real values into probabilities that sum to 1.

**Table 2:** (Alternate antagonism and Paradoxical translation) The alternate antagonism and paradoxical translation recovery patterns seen in two bilingual aphasic subjects; adapted from [10].

	Language (Naming, etc.)	Translation
**First Patient (A.D.)**		
1st Period	Total aphasia	
2nd Period	L1 > L2	
+1 Day	L2 > L1	
+2 Day	L1 > L2	L2 → L1 Bad; L1 → L2 Good
+3 Day	L2 > L1	L2 → L1 Excellent; L1 → L2 Poor
+4 Day	L1 = good	
+11 Day	L2 > L1	L2 → L1 Very poor; L1 → L2 Very poor
+24 Day	L2 ≥ L1	L2 → L1 Poor; L1 → L2 Poor
+25 Day	L2 ≥ L1	L2 → L1 Poor; L1 → L2 Good
**Second Patient**		
1st Week	L1 > L2	
2nd Week	L2 > L1	
3rd Week	L2 ≥ L1	L2 → L1 Excellent; L1 → L2 Very poor
4th Week	L2 = L1	L2 → L1 Excellent; L1 → L2 Excellent
